# Retrospective observational study between robotic thyroidectomy via bilateral axillo-breast approach and conventional open surgery for thyroid cancer

**DOI:** 10.3389/fsurg.2025.1663126

**Published:** 2025-11-20

**Authors:** Fei Kuang, Mengjia Zhou, Weiqin Wang, Wen Tian, Lin Liu

**Affiliations:** 1 Department of Thyroid & Hernia Surgery, Medical Department of General Surgery, Chinese People’s Liberation Army General Hospital, Beijing, China; 2 Department of Ultrasound, Seventh People’s Hospital of Shanghai University of Traditional Chinese Medicine, Shanghai, China

**Keywords:** thyroid cancer, bilateral axillo-breast approach (BABA), da vinci robot, open surgery, lateral neck lymph node dissection

## Abstract

**Background:**

Robotic thyroidectomy has shown good acceptance results and improved cosmetic outcomes. This study aimed to evaluate the safety and efficacy of bilateral axillary-breast approach (BABA) robotic thyroidectomy compared with conventional open surgery for the treatment of thyroid cancer.

**Methods:**

The clinicopathological features and surgical outcomes of 73 papillary thyroid cancer patients treated by robotic surgery using the BABA approach and 62 papillary thyroid cancer patients treated by open surgery in our department from January 2024 to January 2025 were analyzed and compared.

**Results:**

The operation time was longer in the robotic group than in the open surgery group (*P* = 0.001). However, when thyroid cancer requires lymph node dissection in the lateral neck area, the open operation time is longer than that of robotic surgery, although it is no significant (*P* > 0.05). The estimated blood loss was lower in the robotic group than in the open surgery group (*P* < 0.001). Both total number of removed lymph nodes (*P* = 0.019) and metastatic lymph nodes (*P* = 0.002) were higher in the robotic group than in the open surgery group. Postoperative inflammatory reaction was higher in the robotic group than in the open surgery group (*P* < 0.001). No significant difference was observed in the nerve injury or Chyle leakage between the two groups. No recurrence or metastasis was found.

**Conclusions:**

Compared with open surgery, BABA robot radical thyroid cancer surgery with lymph node dissection is safety and efficacy, and have advantages such as less intraoperative blood loss, no neck scar. Especially in lateral cervical lymph node dissection, robotic surgery has obvious advantages.

## Introduction

Thyroid cancer is one of the most common malignant tumors, and its annual incidence rate is on the rise (1, 2). Compared with other malignant tumors, most thyroid cancer patients have a good prognosis, so they not only require complete removal of the lesion, but also hope to have a higher quality of life after surgery (3, 4). Endoscopic surgery is an ideal option for treating thyroid disease because it maintains the aesthetics of the neck. As one of the advanced endoscopic systems, the da Vinci robot has advantages such as 3D high-definition vision and remote control that can filter out operator shaking; it also has flexible internal joints with 7 degrees of freedom, which is more conducive to delicate operations than traditional open surgery (5, 6). Therefore, it has been widely used in thyroid surgery (7–10). Although recent studies have shown that robot-assisted radical thyroidectomy is as safety and efficacy as traditional open surgery (11, 12). However, it is unclear whether robots are superior to open surgery in radical thyroid cancer surgeries of different degrees, especially in lateral neck lymph node dissection.

From January 2024 to January 2025, we treated 73 thyroid cancer patients by robotic surgery via a bilateral axillo-breast approach (BABA) (robotic group). Another 62 thyroid cancer patients who underwent open surgery in the same period served as the control group (open surgery group). The data pertaining to the efficacy and safety of the two groups were analyzed.

## Materials and methods

### Patients

Patients who met the following criteria were included in the study: (1) aged between 18 and 65 years; (2) diagnosed with thyroid cancer by preoperative fine needle aspiration (FNA) or suspected malignant tumors by preoperative FNA examination and confirmed by postoperative pathology; and (3) with a maximum tumor diameter of ≤5 cm as shown by ultrasonography; (4) preoperative nasofibrolaryngoscopy was performedand. The exclusion criteria are as follows: (1) a history of thyroid surgery or radiotherapy in the neck; (2) preoperative examination or intraoperative findings that the tumor has invaded the esophagus, trachea, recurrent laryngeal nerve, or large blood vessels in the neck; (3) preoperative examination showing suspected or clear metastasis to the lateral lymph nodes; (4) preoperative examination showing distant metastasis. The demographic and clinical data showed in Table 1. The sample size of this study was estimated based on the data from a previous pilot study. Choice of surgical approach: All patients were informed of the advantages and disadvantages of robotic surgery and traditional laparotomy and made their own decision before surgery without inducement behavior. All operations were performed by a single surgeon who completed over 1,000 cases of thyroid cancer surgeries using the robotic BABA approach and 5,000 cases of open thyroid cancer surgeries. We analyzed the patients’ medical records and evaluated the surgical outcomes, including the total operative time, estimated intraoperative blood loss, number of resected lymph nodes, number of metastatic lymph nodes, nerve injury, postoperative inflammatory response, and chylous leak, among other data for all patients.

**Table 1:** Demographic and clinical data.

Characteristic	Value
Gender	
Male	45
Female	90
Age (years)^a^	44.00 [36.00, 53.00]
Primary tumor size (cm)^b^	1.54 ± 1.12
Robotic surgery	73
Open surgery	62
Surgical approach	
Unilateral lobectomy + unilateral central node dissection	44
Total thyroidectomy + unilateral central node dissection	13
Total thyroidectomy + bilateral central node dissection	44
Total thyroidectomy + bilateral central node dissection + bilateral Lateral neck node dissection	34

a In deviation distribution, data was presented as median (P25, P75) (p, percentile).

b In normal distribution, data was presented as mean ± standard deviation (SD).

### Open surgery

After satisfactory anesthesia, the patient lies in a supine position, with the shoulder and neck in an overextended position. The surgical area of the neck and shoulder is disinfected and covered with a sterile towel. Two transverse fingers above the suprasternal fossa are made along the skin lines to make a transverse arc-shaped incision about 5–12 cm long. The skin, subcutaneous tissue, and latissimus neck platysma are incised. A free flap is made on the deep surface of the latissimus neck platysma, up to the hyoid level and down to the suprasternal fossa. Cut the anterior cervical fascia along the white line of the neck. Separate the bilateral thyroid tissues from the space between the infrahyoid muscles and the external layer of the thyroid gland. Carefully separate the thyroid gland with an electric knife within the surgical capsule, expose the thyroid gland, inject 0.2 mL of nano-carbon suspension into the thyroid lobe, and cut and ligate the middle thyroid vein. Separate the annular thyroid space, cut and ligate the superior thyroid artery and vein closely at the upper pole of the thyroid gland. Lift the lower pole of the thyroid gland, cut and ligate the blood vessels of the lower pole of the thyroid gland to expose and protect the parathyroid blood supply. Use scissors to free the recurrent laryngeal nerve from the tumor. The electromyographic signals of the recurrent laryngeal nerve and vagus monitored by the nerve monitor show normal responses. Cut the tight tissue between the isthmus of the thyroid gland and the trachea, cut and ligate the papillary ligament of the thyroid gland. Be careful not to damage the site where the recurrent laryngeal nerve enters the larynx. Remove the thyroid gland and isthmus simultaneously. Clear the Lymphoid adipose tissue from below the thyroid cartilage to the clavicle plane, the medial side of the common carotid artery, the area around the recurrent laryngeal nerve, and the pretracheal lymphoid adipose tissue above the sternal incision. The ultrasonic knife was used to free the space between the sternocleidomastoid muscle and the ribbon-like muscle. The right sternocleidomastoid muscle was pulled outward to expose the carotid sheath. The carotid sheath was cut vertically from the surface of the internal jugular vein. The tissues were separated from front to back respectively. The lymphoid adipose tissue in front of the carotid sheath, including the carotid triangle, was lifted from bottom to top, and then this tissue was turned back through the superficial surface of the internal jugular vein. It ascends to the posterior abdomen of the right biceps femoris muscle, exposing and protecting the accessory nerve, and clearing the adipose lymphoid tissue in area II around this segment of the accessory nerve. The ultrasonic scalpel opens the intermuscular system of the sternocleidomastoid muscle, downward to the supraclavicular level and backward to the prevertebral fascia, revealing and protecting the phrenic nerve. The right lymphatic duct was explored at the jugular Angle. The tissues in this area were cut in batches to protect the main trunk of the transverse jugular artery and vein. The entire dissection specimen was separated and dissected backward from the lymph nodes in areas III, IV, and V, and the dissected lymph node specimens were removed.

### Robotic surgery

**Figure 1:**
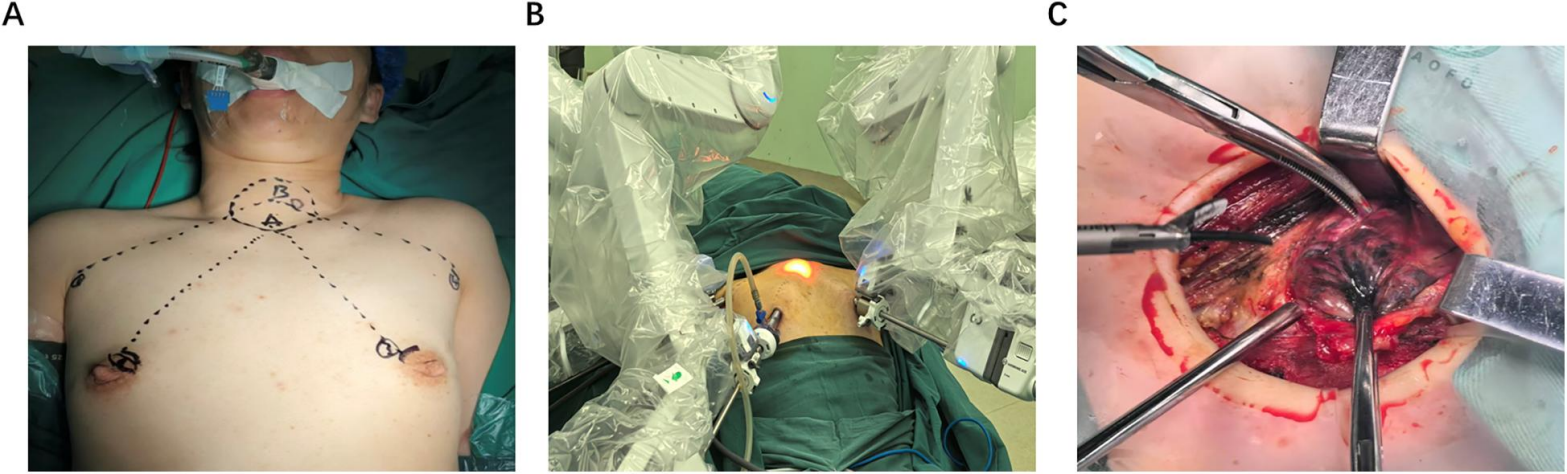
Diagrams of robotic surgery and open surgery approaches for thyroid cancer. **(A)** Drawing instrument arm trajectory lines and working area. **(B)** Each of the four trocars was docked with a robotic arm. **(C)** Open the lateral incision in the neck for surgery to expose the thyroid gland.

**Figure 2:**
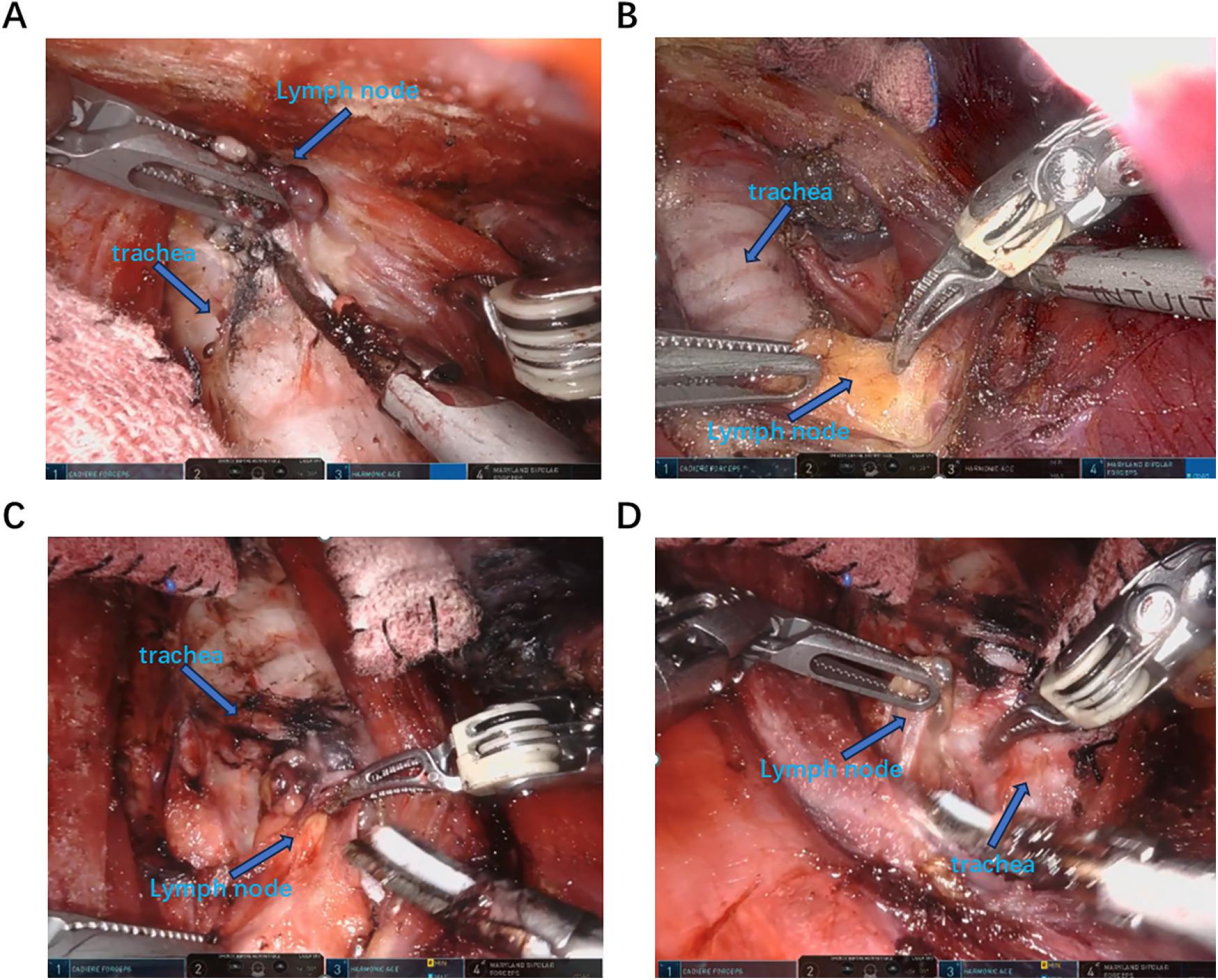
Identify and dissect the lymph nodes in the central area. **(A,B)** The strap muscle was drawn to the left and the trachea was drawn to the right using Maryland and cartier to expose the left median area for central lymph node dissection. **(C,D)** The strap muscle was drawn to the right and the trachea was drawn to the left using Maryland and cartier to expose the right median area for central lymph node dissection.

**Figure 3:**
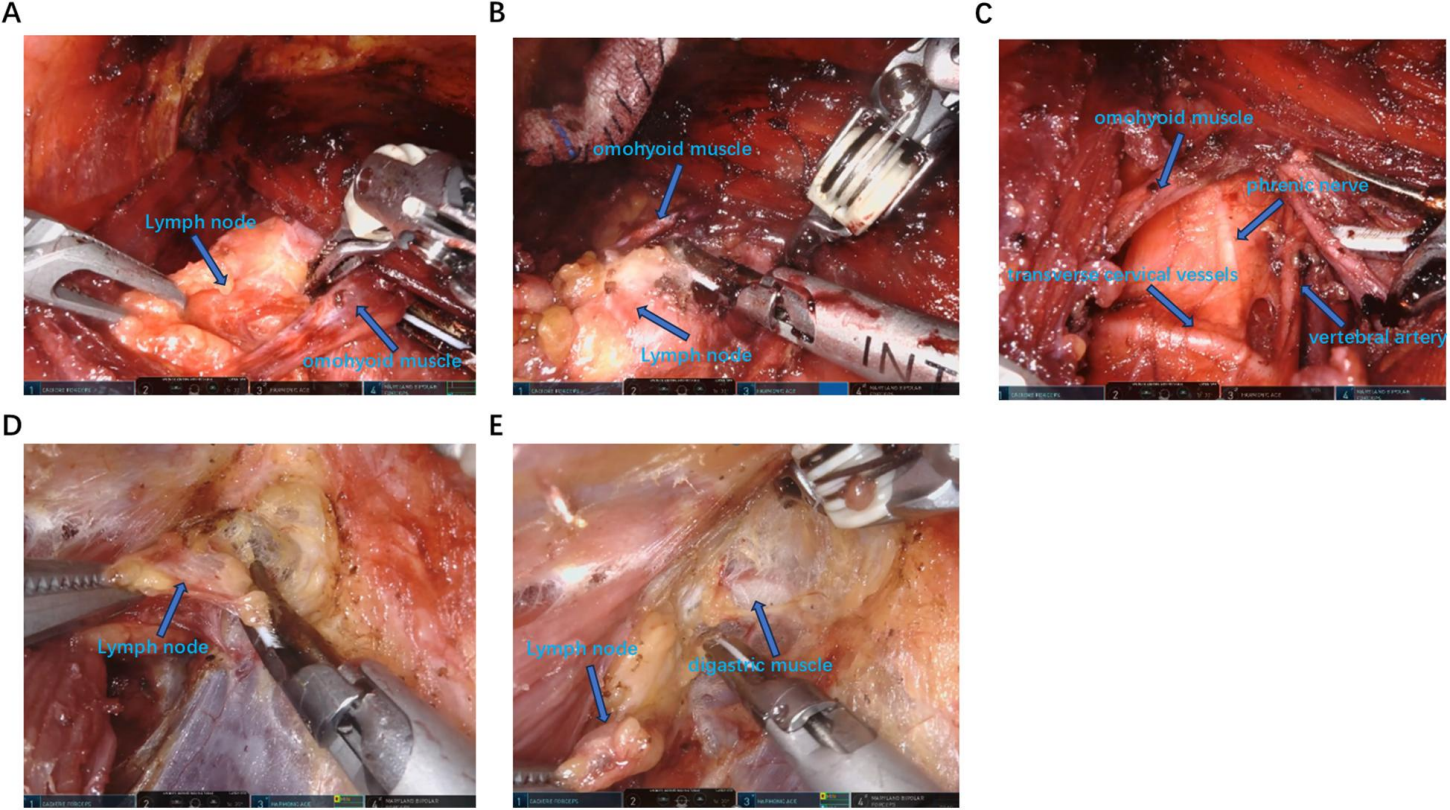
Identify and dissect the lymph nodes in the lateral cervical area. **(A–C)** From the intermuscular space of the sternocleidomastoid muscle to expose the omohyoid muscle and dissect the lymph nodes in area III, IV and V. **(D,E)** Expose the digastric muscle from the inner side of the sternocleidomastoid muscle and dissect the lymph nodes in area II.

After the patient was placed in a supine position with the recurrent laryngeal nerve monitoring tube inserted under general anesthesia, the shoulder was elevated and the neck was extended backward. The routine disinfection and sheet were made. A 1.2 cm long incision was made at two points on the right areola. A long pneumoperitoneum needle was inserted into the suprasternal fossa, and about 50 mL of expansion fluid and 20 mL of air were injected. A separation rod was inserted, and a 12 mm Trocar was placed in the superficial layer of the deep fascia in the chest as the main observation hole. Set the pressure at 8mmHg to establish the CO2 residual cavity. 0.8 cm incisions were made respectively in the left and right axilla and the left areola at 11 points, and an 8 mm Trocar was inserted. The Da Vinci robot is placed in the predetermined position. The 1st, 2nd and 3rd arms are respectively connected to the ultrasonic scalpel, the gripper and the Maryland forceps (Figure 1). A latent separated flap is made between the deep surface of the latissimus neck platysma and the deep cervical fascia, reaching up to the thyroid cartilage, down to the supraspinal fossa, and on both sides to the anterior edge of the sternocleidomastoid muscle. Cut the white line of the neck to reach the thyroid capsule, separate the anterior cervical muscle groups on both sides, and expose the thyroid gland. The bilateral surgical capsules were carefully separated with an ultrasonic scalpel to expose the bilateral lobules of the thyroid gland. The nano-carbon suspension was percutaneous injected at the neck with 0.2 mL under the capsule of the bilateral lobules of the thyroid gland. Subsequent operations were carried out after the nano-carbon black staining diffused uniformly. Expose the thyroid gland, cut and ligate the middle thyroid vein, separate the cricothyroid space, use a surgical clip to ligate and cut the superior thyroid artery and vein close to the thyroid at the upper pole of the thyroid gland, lift the lower pole of the thyroid gland, cut and ligate the blood vessels at the lower pole of the thyroid gland, expose and protect the parathyroid blood supply, release the recurrent laryngeal nerve, and the electromyographic signals of the recurrent laryngeal nerve and vagus nerve monitored by a nerve monitor showed normal responses. Cut the tight tissue between the isthmus of the thyroid gland and the trachea, cut and ligate the papillary ligament of the thyroid gland, and be careful not to damage the site where the recurrent laryngeal nerve enters the larynx. Remove both the thyroid gland and the isthmus at the same time, and clear the lymphoid adipose tissue below the thyroid cartilage to the clavicle plane, the medial side of the common carotid artery, the lymphoid adipose tissue around the recurrent laryngeal nerve, and the lymphoid adipose tissue in front of the trachea above the sternal incision (Figure 2). The ultrasonic knife is used to free the space between the sternocleidomastoid muscle and the band-shaped muscle. The sternocleidomastoid muscle is pulled outward to expose the carotid sheath. Make a longitudinal incision along the surface of the internal jugular vein to open the carotid sheath. The tissues are separated from the front to the back respectively. The lymphoid adipose tissue in front of the carotid sheath, including the carotid triangle, is lifted from bottom to top. Then, this tissue is flipped back through the superficial surface of the internal jugular vein and released upward to the posterior abdomen of the biceps. Expose and protect the accessory nerve, and clear the adipose lymphoid tissue in area II around this segment of the accessory nerve. The intermuscular system of the sternocleidomastoid muscle was opened with an ultrasonic knife, moving downward to the supraclavicular level and backward to the prevertebral fascia to expose and protect the phrenic nerve. Lymphatic ducts were detected at the jugular Angle. The tissues in this area were cut in batches to protect the main trunk of the transverse carotid artery and vein. The entire dissection specimen was separated and dissected backward from the lymph nodes in areas III, IV, and V, and the dissected lymph node specimens were removed (Figure 3).

### Postoperative treatment and follow-up

The patient's voice was observed postoperatively. The color and volume of the drainage fluid were recorded daily, and the drainage tube was removed when the drainage fluid became light red and the daily volume was less than 15 mL. Patients were followed up in the clinic at 1, 3, and 6 months after surgery and every 6 months thereafter. Follow-up evaluations included thyroid function tests, thyroglobulin (TG) measurements, serum calcium and parathyroid hormone (PTH) levels, and neck ultrasound. Based on laboratory test results, a serum calcium level of less than 2.2 mmol/L was defined as hypocalcemia. A parathyroid hormone (PTH) level of less than 15 pg/mL was defined as hypoparathyroidism. If PTH returned to normal levels within 6 months, the patient was diagnosed with transient hypoparathyroidism, otherwise permanent hypoparathyroidism. Any bleeding requiring surgical intervention was considered postoperative bleeding.

### Statistical analysis

For descriptive statistics of quantitative variables, mean ± standard deviation (SD) and range were used to describe central tendency and dispersion. For analysis of the differences in proportions, Chi-square test was used. Fisher's exact test was used if the assumptions of Chi-square test were violated. Independent samples *t*-test was used to compare the level of quantitative variables between the two groups. Data were analyzed using SPSS 15.0 on Windows (SPSS Inc., Chicago, IL, USA). A *P* < 0.05 was considered statistically significant.

## Results

### Overall comparison of the two groups

No significant differences were observed in patient's age, gender, size of tumor, Nerve injury (intraoperative nerve detection and postoperative voice changes), Chyle leakage (color of the postoperative drainage fluid) between the open surgery group and the robotic group. The operation time (initial incision to the suturing of the approach) was longer in the robotic group than in the open surgery group (*P* = 0.001). The estimated blood loss (reference anesthesia record) was lower in the robotic group than in the open surgery group (*P* < 0.001). Both total number of removed lymph nodes (*P* = 0.019) and metastatic lymph nodes (postoperative pathological results) (*P* = 0.002) were higher in the robotic group than in the open surgery group. Postoperative inflammatory reaction [postoperative check white blood cell (WBC) and C-reactive protein (CRP)] was higher in the robotic group than in the open surgery group (*P* < 0.001) (Table 2).

**Table 2:** Overall comparison of the two groups.

Items	Robotic group (*n* = 73)	Open surgery group (*n* = 62)	*P* value
Age (years)	42.00 [34.00, 52.00]	44.00 [36.00, 53.00]	0.366
Gender			0.002
Male	20 (17.7)	25 (40.3)	
Female	53 (82.3)	37 (59.7)	
Primary tumor size (cm)	1.00 [0.70, 1.50]	1.25 [0.80, 2.00]	0.025
Surgical approach			0.055
Unilateral lobectomy + unilateral central node dissection	23 (38.1)	21 (33.9)	
Total thyroidectomy + unilateral central node dissection	7 (6.2)	6 (9.7)	
Total thyroidectomy + bilateral central node dissection	27 (41.6)	17 (27.4)	
Total thyroidectomy + bilateral central node dissection + bilateral Lateral neck node dissection	16 (14.2)	18 (29.0)	
Operating time (min)	90.00 [78.00, 115.00]	70.00 [50.50, 108.25]	0.001
Estimated blood loss (ml)	15.00 [15.00, 15.00]	25.00 [15.00, 25.00]	<0.001
Metastatic lymph nodes	2.50 [0.00, 8.75]	0.00 [0.00, 3.00]	0.002
Total number of removed lymph nodes	10.50 [5.00, 20.75]	7.00 [4.00, 12.00]	0.019
Nerve injury			0.228
Yes	5 (4.4)	0 (0.0)	
No	68 (95.6)	62 (100.0)	
Postoperative inflammatory reaction			<0.001
Yes	40 (53.1)	15 (24.2)	
No	33 (46.9)	47 (75.8)	
Chyle leakage			0.493
Yes	3 (2.7)	0 (0.0)	
No	70 (97.3)	62 (100.0)	

### Comparison of unilateral lobectomy + unilateral central node dissection in the two groups

No significant differences were observed in patient's age, gender, size of tumor, Nerve injury, Chyle leakage, total number of removed lymph nodes, metastatic lymph nodes between the open surgery group and the robotic group. The operation time was longer in the robotic group than in the open surgery group (*P* = 0.001). The estimated blood loss was lower in the robotic group than in the open surgery group (*P* < 0.001) (Table 3).

**Table 3:** Comparison of unilateral lobectomy + unilateral central node dissection in the two groups.

Items	Robotic group (*n* = 23)	Open surgery group (*n* = 21)	*P* value
Unilateral lobectomy + unilateral central node dissection	23 (38.1)	21 (33.9)	
Age (years)	44.00 [33.50, 50.50]	42.00 [37.00, 57.00]	0.577
Gender			0.203
Male	6 (14.0)	11 (52.4)	
Female	17 (86.0)	10 (47.6)	
Primary tumor size (cm)	0.80 [0.65, 1.50]	1.00 [0.70, 2.00]	0.505
Operating time (min)	80.00 [65.00, 90.00]	50.00 [40.00, 80.00]	0.001
Estimated blood loss (ml)	15.00 [10.00, 15.00]	25.00 [15.00, 25.00]	<0.001
Metastatic lymph nodes	0.00 [0.00, 1.00]	0.00 [0.00, 1.00]	0.233
Total number of removed lymph nodes	5.00 [3.00, 7.00]	5.00 [2.00, 7.00]	0.857
Nerve injury			1
Yes	1 (2.3)	0 (0.0)	
No	22 (97.7)	21 (100.0)	
Postoperative inflammatory reaction			0.079
Yes	7 (39.5)	3 (14.3)	
No	16 (60.5)	18 (85.7)	
Chyle leakage			1
Yes	1 (2.3)	0 (0.0)	
No	22 (97.7)	21 (100.0)	

### Comparison of total thyroidectomy + unilateral central node dissection in the two groups

No significant differences were observed in patient's age, gender, size of tumor, Nerve injury, Chyle leakage, total number of removed lymph nodes, metastatic lymph nodes, estimated blood loss between the open surgery group and the robotic group. The operation time was longer in the robotic group than in the open surgery group (*P* = 0.003) (Table 4).

**Table 4:** Comparison of total thyroidectomy + unilateral central node dissection in the two groups.

Items	Robotic group (*n* = 7)	Open surgery group (*n* = 6)	*P* value
Total thyroidectomy + unilateral central node dissection	7 (6.2)	6 (9.7)	
Age (years)	55.00 [35.00, 59.50]	43.50 [39.25, 50.75]	0.943
Gender			1
Male	2 (28.6)	1 (16.7)	
Female	5 (71.4)	5 (83.3)	
Primary tumor size (cm)	1.30 [1.10, 1.40]	1.35 [0.62, 2.22]	0.892
Operating time (min)	101.00 [92.50, 115.00]	60.00 [60.00, 60.00]	0.003
Estimated blood loss (ml)	15.00 [15.00, 15.00]	25.00 [17.50, 25.00]	0.285
Metastatic lymph nodes	2.00 [0.00, 4.75]	0.00 [0.00, 0.50]	0.221
Total number of removed lymph nodes	8.00 [4.25, 11.75]	1.00 [1.00, 6.50]	0.168
Nerve injury			1
Yes	0 (0.0)	0 (0.0)	
No	7 (100.0)	6 (100.0)	
Postoperative inflammatory reaction			0.157
Yes	5 (71.4)	1 (16.7)	
No	2 (28.6)	5 (83.3)	
Chyle leakage			1
Yes	0 (0.0)	0 (0.0)	
No	7 (100.0)	6 (100.0)	

### Comparison of total thyroidectomy + bilateral central node dissection in the two groups

No significant differences were observed in patient's age, gender, size of tumor, Nerve injury, Chyle leakage, total number of removed lymph nodes, metastatic lymph nodes between the open surgery group and the robotic group. The operation time was longer in the robotic group than in the open surgery group (*P* < 0.001). The estimated blood loss was lower in the robotic group than in the open surgery group (*P* < 0.001). Postoperative inflammatory reaction was higher in the robotic group than in the open surgery group (*P* = 0.017) (Table 5).

**Table 5:** Comparison of total thyroidectomy + bilateral central node dissection in the two groups.

Items	Robotic group (*n* = 27)	Open surgery group (*n* = 17)	*P* value
Total thyroidectomy + bilateral central node dissection	27 (41.6)	17 (27.4)	
Age (years)	42.00 [36.50, 52.00]	47.00 [42.00, 52.00]	0.386
Gender			0.974
Male	9 (19.1)	4 (23.5)	
Female	18 (80.9)	13 (76.5)	
Primary tumor size (cm)	0.90 [0.55, 1.50]	1.00 [0.80, 1.50]	0.463
Operating time (min)	90.00 [80.00, 110.00]	70.00 [60.00, 70.00]	<0.001
Estimated blood loss (ml)	15.00 [15.00, 15.00]	25.00 [15.00, 25.00]	<0.001
Metastatic lymph nodes	1.00 [0.00, 5.00]	0.00 [0.00, 2.00]	0.165
Total number of removed lymph nodes	11.00 [7.00, 13.00]	8.00 [5.00, 12.50]	0.143
Nerve injury			0.511
Yes	4 (8.5)	0 (0.0)	
No	23 (91.5)	17 (100.0)	
Postoperative inflammatory reaction			0.017
Yes	16 (55.3)	3 (17.6)	
No	11 (44.7)	14 (82.4)	
Chyle leakage			0.959
Yes	2 (4.3)	0 (0.0)	
No	25 (95.7)	17 (100.0)	

### Comparison of total thyroidectomy + bilateral central node dissection + bilateral lateral neck node dissection in the two groups

No significant differences were observed in patient's age, gender, size of tumor, Nerve injury, Chyle leakage, total number of removed lymph nodes, metastatic lymph nodes between the open surgery group and the robotic group. The estimated blood loss was lower in the robotic group than in the open surgery group (*P* = 0.002). However, only when thyroid cancer requires lymph node dissection in the lateral neck area, the open operation time is longer than that of robotic surgery, although it is no significant (*P* > 0.05) (Table 6).

**Table 6:** Comparison of total thyroidectomy + bilateral central node dissection + bilateral lateral neck node dissection in the two groups.

Items	Robotic group (*n* = 16)	Open surgery group (*n* = 18)	*P* value
Total thyroidectomy + bilateral central node dissection + bilateral Lateral neck node dissection	16 (14.2)	18 (29.0)	
Age (years)	32.50 [28.00, 44.75]	39.00 [32.25, 50.75]	0.317
Gender			0.123
Male	3 (18.8)	9 (50.0)	
Female	13 (81.2)	9 (50.0)	
Primary tumor size (cm)	1.30 [0.98, 1.55]	1.50 [1.35, 2.45]	0.046
Operating time (min)	135.00 [123.00, 165.00]	140.00 [120.00, 159.75]	0.654
Estimated blood loss (ml)	15.00 [15.00, 15.00]	25.00 [15.00, 25.00]	0.002
Metastatic lymph nodes	11.00 [9.25, 16.50]	6.50 [4.00, 12.75]	0.195
Total number of removed lymph nodes	31.50 [23.75, 38.50]	23.00 [20.00, 33.75]	0.269
Nerve injury			1
Yes	0 (0.0)	0 (0.0)	
No	16 (100.0)	18 (100.0)	
Postoperative inflammatory reaction			0.145
Yes	12 (75.0)	8 (44.4)	
No	4 (25.0)	10 (55.6)	
Chyle leakage			1
Yes	0 (0.0)	0 (0.0)	
No	16 (100.0)	18 (100.0)	

## Discussion

The da Vinci robotic system has been used for surgical treatment of thyroid cancer (13) because of its high-definition 3D field of view, remote-controlled operation to eliminate hand tremors, and internally articulated instruments (14, 15). However, whether robotic surgery is superior to traditional open surgery remains controversial due to its high cost and concerns about clinical outcomes (16–18). It is worth recognizing that this is the first study to compare BABA robotic surgery with conventional open surgery, and our results show that BABA robotic thyroidectomy can maintain the aesthetic appearance of the neck and has significant results.

Traditionally, the incision has been enlarged into a larger transverse incision to complete the required neck resection. Since the front of the neck is prominent and a frequently exposed part of the body, this may leave an unsightly neck scar, which can be a nuisance to patients. In addition, most thyroid cancer patients are young women who prefer to avoid scarring on their necks. Therefore, surgeons have gone to great lengths to address this scarring issue. In 1997, the first endoscopic thyroidectomy was performed (19). In 2009, the feasibility of robotic thyroidectomy via the axillary approach was demonstrated, which further improved the aesthetic outcome of the surgery (20). To our knowledge, several improved approaches have been reported in the literature, which can be roughly divided into the following categories: smaller neck incisions, including incisions in natural body cavities or body surfaces; more distal incisions, such as chest wall, periauricular, axillary, retroauricular, and transoral incisions; and combined incisions. All surgical approaches have their own advantages and disadvantages. For example, the axillary approach has limitations in reaching the contralateral central lymph nodes (21, 22). The BABA procedure provides a complete and symmetrical surgical field of view of anatomical structures (such as the superior and inferior thyroid vessels, recurrent laryngeal nerve, parathyroid glands, and trachea), enables exploration of both thyroid glands, provides more space for instrument manipulation, and enables the removal of larger nodules. No brachial plexus injury or axillary skin flap perforation occurred during the BABA procedure (13, 22, 23). Robotic total thyroidectomy with neck dissection via BABA had similar complication rates as open procedure. In terms of clinical safety and efficacy, robotic total thyroidectomy with neck lymph node dissection is equivalent to open surgery. The small scars in the bilateral axilla and nipple areola were almost invisible. However, some young women may refuse to undergo this procedure because the BABA procedure involves their breasts. Further research is needed to explore other better methods to minimize this disadvantage (24, 25).

As with any emerging treatment technology, careful patient selection is critical. While it is important to tailor the surgical approach to the patient's concerns and expectations, it is always imperative to adhere to basic surgical oncology principles. The oncological safety is more important than the cosmetic demand. We analyzed the clinical data of 73 thyroid cancer patients who underwent robotic surgery using the BABA approach and 62 thyroid cancer patients who underwent conventional open surgery. The results showed that there are no significant differences were observed in patient's age, gender, size of tumor, Nerve injury, Chyle leakage between the open surgery group and the robotic group. The operation time was longer in the robotic group than in the open surgery group (*P* = 0.001). The estimated blood loss was lower in the robotic group than in the open surgery group (*P* < 0.001). Both total number of removed lymph nodes (*P* = 0.019) and metastatic lymph nodes (*P* = 0.002) were higher in the robotic group than in the open surgery group. Postoperative inflammatory reaction was higher in the robotic group than in the open surgery group (*P* < 0.001), because the BABA robotic approach for thyroid cancer radical surgery requires the establishment of an operating space under the chest skin, this might be the main cause of postoperative inflammatory reaction. The long operation time of thyroid cancer surgery using the BABA robotic approach might also be a factor contributing to the postoperative inflammatory response. We checked the inflammatory response through postoperative white blood cell (WBC) and C-reactive protein (CRP) levels, the surgical stress response can also lead to an increase in postoperative WBC and CRP levels. Although recent studies have shown that robot-assisted radical thyroidectomy is no different from traditional open surgery in terms of safety and effectiveness (11, 12). However, it is unclear whether robots are superior to open surgery in radical thyroid cancer surgeries of different degrees, especially in lateral neck lymph node dissection. We grouped the surgeries for thyroid cancer of different degrees, The robot has obvious advantages in Total thyroidectomy + bilateral central node dissection + bilateral Lateral neck node dissection, compared with other degrees of thyroid cancer, the robotic surgery time for this thyroid cancer is shorter than that for open surgery, although it is no significant (*P* > 0.05). This fully reveals the safety and efficacy of robot-based radical thyroidectomy, especially in the lymph node dissection of the lateral neck region, where the advantages of robot-based surgery are obvious.

Studies have shown that the learning curve for performing robotic thyroid surgery requires more than 50 surgical experiences (26–29). However, this surgeon should be more experienced and skilled in handling complex situations in thyroid surgery. The limitations of this study are as follows: (I) To avoid the influence of the surgeon's proficiency on the surgical outcomes, we selected patients who were operated on by the most experienced surgeons in both the robotic surgery group and the open surgery group. Even so, the selection bias is inevitable. (II) Our study was a single-center clinical trial with a small sample size, so more multicenter randomized controlled studies are needed for further verification. (III) Although the prognosis of thyroid cancer is generally good after surgical treatment, the effectiveness of tumor radicalization is evaluated based on the postoperative pathological lymph node metastasis situation. However, the assessment of tumor safety mainly relies on long-term postoperative follow-up. This study did not adequately evaluate the safety of the tumor due to the short follow-up period for the patients after surgery. In the future, longer follow-up periods are needed. In the future, we will conduct a large-sample retrospective study in multiple centers to further determine the superiority and safety of robotic thyroid cancer surgery.

## Conclusion

The priority of any surgical procedure is to ensure patient safety with the best patient outcomes. This study demonstrates that BABA robot radical thyroid cancer surgery with lymph node dissection is safe and effective, and have advantages such as less intraoperative blood loss, no neck scar. Especially in lateral cervical lymph node dissection, robotic surgery has obvious advantages.

## Data Availability

The original contributions presented in the study are included in the article/Supplementary Material, further inquiries can be directed to the corresponding author.
